# Signaling Pathways from the Endoplasmic Reticulum and Their Roles in Disease

**DOI:** 10.3390/genes4030306

**Published:** 2013-07-01

**Authors:** Hisae Kadowaki, Hideki Nishitoh

**Affiliations:** Laboratory of Biochemistry and Molecular Biology, Department of Medical Sciences, University of Miyazaki, 5200 Kihara, Kiyotake, Miyazaki 889-1692, Japan; E-Mail: kadowaki@med.miyazaki-u.ac.jp

**Keywords:** ER stress, unfolded protein response (UPR), apoptosis, diseases

## Abstract

The endoplasmic reticulum (ER) is an organelle in which newly synthesized secretory and transmembrane proteins are assembled and folded into their correct tertiary structures. However, many of these ER proteins are misfolded as a result of various stimuli and gene mutations. The accumulation of misfolded proteins disrupts the function of the ER and induces ER stress. Eukaryotic cells possess a highly conserved signaling pathway, termed the unfolded protein response (UPR), to adapt and respond to ER stress conditions, thereby promoting cell survival. However, in the case of prolonged ER stress or UPR malfunction, apoptosis signaling is activated. Dysfunction of the UPR causes numerous conformational diseases, including neurodegenerative disease, metabolic disease, inflammatory disease, diabetes mellitus, cancer, and cardiovascular disease. Thus, ER stress-induced signaling pathways may serve as potent therapeutic targets of ER stress-related diseases. In this review, we will discuss the molecular mechanisms of the UPR and ER stress-induced apoptosis, as well as the possible roles of ER stress in several diseases.

## 1. Introduction

Secretory and transmembrane proteins translocate into the endoplasmic reticulum (ER) through either co-translational or post-translational approaches. These ER-translocated proteins are modified and folded by ER chaperones and folding factors in order to form their proper tertiary structures. Correctly folded proteins exit the ER and are either targeted to the membrane or released from the cell surface through the secretory pathway [1]. Protein folding, modification, and trafficking within the ER are strictly regulated by the protein quality control system.

The functions of the ER are disrupted by various intracellular and extracellular stimuli, which results in so-called ER stress, and this condition can be triggered by the inhibition of glycosylation, reduction of disulfide bonds, depletion of ER calcium stores, impairment of protein transport to the Golgi, increased ER protein synthesis, impairment of ER-associated degradation (ERAD), or the expression of mutated ER proteins. Under ER stress conditions, unfolded proteins accumulate in the ER, and this buildup eventually induces the perturbation of cellular activities. The resulting fate of the cell is either survival or apoptosis, depending on the cellular response to the stress. When misfolded, proteins accumulate in the ER lumen, cells activate a self-protective mechanism, termed the unfolded protein response (UPR), to survive the ER stress conditions. The UPR of eukaryotic cells consists of three different mechanisms: (1) translational attenuation to limit further protein loads [2], (2) transcriptional activation of genes encoding factors involved in ER protein folding and degradation [3], and (3) ERAD, which serves to restore the folding capacity through the clearance of unfolded or misfolded proteins by retrotranslocating these proteins from the ER into the cytosol via the ubiquitin-proteasome system [4].

**Figure 1:**
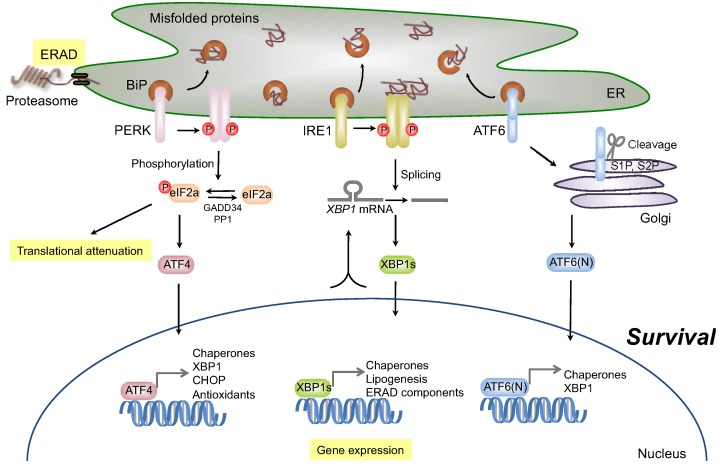
Survival signaling under endoplasmic reticulum (ER) stress conditions. The accumulation of misfolded proteins activates three ER stress sensors: activating transcription factor-6 (ATF6), inositol-requiring transmembrane kinase/endoribonuclease 1 (IRE1), and double-stranded RNA-dependent protein kinase (PKR)-like eukaryotic initiation factor 2α (eIF2α) kinase (PERK). ATF6 is activated following cleavage with S1P and S2P, after transport to the Golgi. Activated ATF6 (ATF6(N)) functions as a transcription factor and induces the expression of ER chaperones and XBP1. Activated IRE1 induces the splicing of *XBP1* messenger RNA (mRNA), and the resulting spliced XBP1 protein (XBP1s) translocates to the nucleus and controls the transcription of ER-resident chaperones and genes involved in lipogenesis and ER-associated degradation (ERAD). The activated PERK subsequently blocks general protein synthesis by phosphorylation of eIF2α, which enables the translation of eIF2α-activating transcription factor-4 (ATF4). ATF4 then translocates to the nucleus and induces the transcription of many genes required for ER quality control.

**Figure 2:**
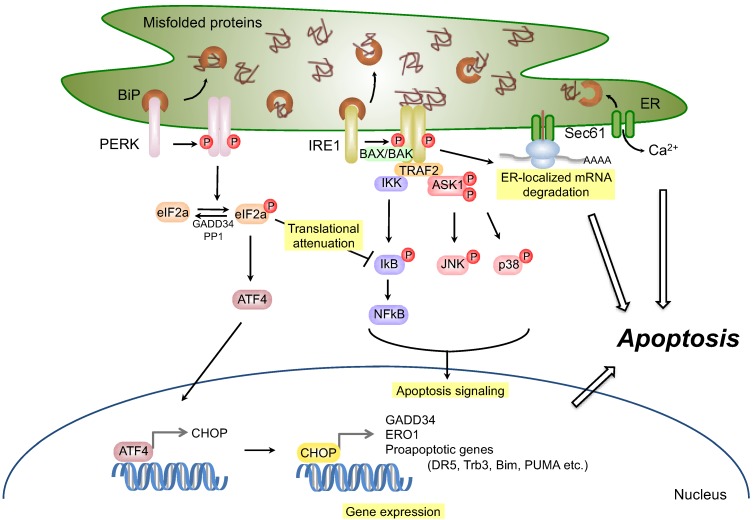
Apoptosis signaling under ER stress conditions. Prolonged or severe ER stress, as well as dysfunction of the unfolded protein response (UPR), induces apoptosis signaling, primarily through the IRE1 and PERK pathways. In the IRE1 pathway, activated IRE1 recruits TRAF2 and ASK1 on the ER membrane and activates the ASK1-dependent apoptosis pathway. In addition, the IKK-NFκB pathway is also activated by IRE1-TRAF2 and induces an apoptotic response. Proapoptotic Bcl-2 family members, Bax and Bak, interact with IRE1 and promote its RNase/kinase activity. Moreover, IRE1 induces ER-localized mRNA degradation. In the PERK pathway, ATF4 induced by the PERK-eIF2α pathway upregulates the expression of CHOP, which in turn activates the transcription of GADD34, ER oxidoreductase 1 (ERO1), and many proapoptotic factors. GADD34 then promotes dephosphorylation of eIF2α with PP1, canceling translational attenuation, and leads to an increase of protein loads into the ER. Furthermore, the translational attenuation of global proteins by the PERK-eIF2α pathway also applies to IκB, which has been terminally linked to the activation of NFκB.

In mammals, the UPR signaling pathway is initiated by three ER membrane-associated sensors: activating transcription factor-6 (ATF6), inositol-requiring transmembrane kinase/endoribonuclease 1 (IRE1), and double-stranded RNA-dependent protein kinase (PKR)-like eukaryotic initiation factor 2α (eIF2α) kinase (PERK) (Figure 1). If the survival signal is insufficient to relieve the cells from ER stress, cells may undergo apoptosis to destroy ER stress-damaged cells. Many reports have shown that several molecules, including IRE1 [5,6], apoptosis signal-regulating kinase 1 (ASK1) [7], Bax/Bak [8,9,10], PERK, eIF2α-activating transcription factor-4 (ATF4) [11], and CCAAT enhancer-binding protein (C/EBP) homologous protein (CHOP, also known as a growth arrest- and DNA damage-inducible gene 153 (GADD153)) [12,13], are related to ER stress-induced apoptosis signaling pathways (Figure 2). Dysfunction of the UPR, or prolonged ER stress, disrupts ER homeostasis. A large number of groups have described the relation between ER stress responses and a variety of human diseases, including neurodegenerative disease, metabolic disease, inflammatory disease, diabetes mellitus, cancer, and cardiovascular disease. Therefore, it is important to understand the role of the UPR in the pathogenesis of these diseases. In this review, we summarize the molecular mechanisms of ER stress-induced survival and apoptosis signaling pathways and discuss the possibility that UPR signaling components could serve as potent therapeutic targets for the treatment of diseases.

## 2. The Signaling Pathways from Three ER Stress Sensors during the UPR

### 2.1. Signaling through Activating Transcription Factor-6 (ATF6)

ATF6 is a basic leucine zipper protein (bZIP)-containing transcription factor and a type II ER transmembrane protein. In mammals, there are two *ATF6* genes, *ATF6α* and *ATF6β*. The ATF6α and ATF6β proteins are ubiquitously expressed [14]. In response to the accumulation of misfolded proteins in the ER, immunoglobulin-binding protein (BiP), which is an ER chaperone also known as glucose-regulated protein 78 (GRP78), dissociates from ATF6, leading to the interaction of ATF6 with misfolded proteins [15]. In turn, ATF6 translocates from the ER membrane to the Golgi [16], where it is processed by site-1 protease (S1P) and site-2 protease (S2P) in the luminal domain and transmembrane domain, respectively. As a result, an *N*-terminal cytosolic domain of ATF6 [ATF6(N)], which contains a bZIP domain, translocates to the nucleus and induces a number of UPR target genes, including ER chaperones {e.g., *BiP*, *protein disulfide isomerase* (*PDI*), and *GRP94*} and *X-box-binding protein 1* (*XBP1*), which eventually induces ERAD components, {e.g., ER degradation enhancing alpha-mannosidase-like protein (EDEM), yeast Der1 like protein (Derlin), HRD1 (mammalian homolog of yeast HMG-CoA reductase degradation 1 protein (Hrd1p)), SEL1L (mammalian homolog of Hrd3p), and homocysteine-induced endoplasmic reticulum protein (Herp)} [3,17,18,19,20,21] (Figure 1). Although *ATF6α^−/−^* and *ATF6β^−/−^* mice are viable, double-knockout mice of *ATF6α* and *ATF6β* are embryonic lethal, suggesting that ATF6α and ATF6β compensate for each other in early development [22]. To resolve their specific functions in organelles, further detailed research will be required.

Recent studies have shown that some bZIP transcription factors are found in the ER transmembrane area in a tissue-specific manner and are activated by intramembrane proteolysis. This phenomenon is similar to that of ATF6 {e.g., cAMP responsive element-binding protein H (CREBH/CREB3L3) in hepatocytes, the pyloric stomach, and the small intestine [23]; OASIS (CREB3L1) in astrocytes and osteoblasts [24]; Tisp40/AIbZIP/CREB3L4/CREB4 in the testis [25,26]; and BBF2H7/CREB3L2 [27] and Luman/LZIP/CREB3 [28,29], which are expressed ubiquitously}. These ATF6-like molecules may possess specialized functions for controlling the UPR signaling pathway in specific tissues. Additional studies are needed to understand their specific physiological roles.

### 2.2. Signaling through Inositol-Requiring Transmembrane Kinase/Endoribonuclease 1 (IRE1)

IRE1 is a type I ER transmembrane protein with serine/threonine kinase activity and endoribonuclease (RNase) activity in its *C*-terminal cytosolic domain [30,31], and is conserved from yeast to humans. There is only one ER stress receptor in yeast, which is termed Ire1p. Yeast genetic studies contributed to the identification of several key factors that are involved in the UPR, such as Hac1p (a downstream target of Ire1p) [32,33]. Activated Ire1p induces the unconventional cytosolic splicing of *Hac1p* messenger RNA (mRNA), resulting the induction of ER chaperones. There are two mammalian homologs of yeast Ire1p. IRE1α is expressed ubiquitously and IRE1β is expressed only in intestinal epithelial cells [34]. 

#### 2.2.1. Survival Signaling via IRE1

Activation of IRE1 is triggered by the dissociation of BiP from the luminal domain of IRE1. The misfolded proteins that accumulate in the ER associate with the ER luminal domain of IRE1α, leading to oligomerization, autophosphorylation of its kinase domain, and finally, activation of the RNase domain of IRE1α in response to the conformational change [35,36]. As well as yeast Ire1p-Hac1p pathway, activated IRE1α also induces the unconventional cytosolic splicing of *XBP1* mRNA to express a potent transcriptional factor, XBP1s, as a product of the translational frameshift [37]. In turn, XBP1s upregulates the transcription of UPR target genes linked to protein folding, quality control, protein secretion, and ERAD [38] (Figure 1).

Deletion of *IRE1α* and *XBP1* causes embryonic lethality [6,34,39], suggesting that IRE1α and XBP1 are required for development. Although a recent study has revealed that IRE1α plays an essential role in placental development and promotes embryonic viability [40], the question of whether XBP1 is involved in placental development remains unanswered. In addition, studies using a cell-specific deletion of *XBP1* have shown that XBP1 is required for the terminal differentiation of B-lymphocytes to plasma cells [41], and that the IRE1α-XBP1 signaling pathway is required for pancreatic β cell function [42] (see Section 4.4). Although further investigation is needed, it is likely that other downstream pathways under the activation of IRE1α, and not only the XBP1 pathway, may exist in some specific tissues. In contrast, *IRE1β^−/−^* mice develop normally but exhibit increased susceptibility to experimentally induced colitis, and this phenotype is consistent with the intestinal epithelium-specific expression of this protein [43] (see Section 4.3).

#### 2.2.2. Apoptosis Signaling via IRE1

During prolonged ER stress, activated IRE1 interacts with tumor necrosis factor receptor associated factor 2 (TRAF2) through its cytosolic domain [6]. The IRE1-TRAF2 complex recruits and activates ASK1, which is also known as MAP kinase kinase kinase, leading to activation of JNK pathway [7]. ER stress-induced activation of the ASK1-JNK pathway triggers apoptosis [7]. In addition, several proapoptotic Bcl-2 family members such as BAX/BAK interact with IRE1α and upregulate its RNase/kinase activity, resulting in the splicing of *XBP1* mRNA, transcriptional activation of XBP1 target genes, JNK phosphorylation, and apoptosis [8]. These findings suggest that a complex composed of IRE1α and its associated factors may play important roles in apoptosis under severe or prolonged ER stress conditions (Figure 2).

In addition to the splicing of *XBP1* mRNA for maintaining ER functions, activated IRE1α causes the decay of ER-localized mRNAs, which encode ER homeostatic factors in *Drosophila* and mammals [44,45,46] (Figure 2). Although it is still controversial whether IRE1-mediated mRNA decay contributes to the maintenance or disturbance of ER homeostasis, the intense activation of IRE1 RNase may trigger the apoptosis pathway. Sustained activation of IRE1α also causes the rapid decay of select microRNAs, which suppress the translation of *caspase-2* mRNA and thus results in markedly elevated protein levels of this initiator protease in the mitochondrial apoptosis pathway [47]. These observations suggest that IRE1α regulates translation of a proapoptotic protein by terminating microRNA biogenesis and that the regulation of noncoding RNA expression levels is a part of ER stress responses. The RNase activity of IRE1α may be a double-edged sword, as it consists of both the splicing of *XBP1* mRNA in the UPR and the decay of ER homeostatic mRNAs and microRNAs to induce apoptosis (Figure 2). Future investigations will be needed to understand the mechanisms by which these two functions of IRE1 are regulated. 

It has been reported that cyclin-dependent kinase 5 (CDK5) and MEKK1 (also known as MAPKKK) mediate an ER stress-induced apoptosis signaling pathway in a *Drosophila* model of retinitis pigmentosa, which is an autosomal dominant disorder [48]. During ER stress, CDK5 phosphorylates MEKK1, which subsequently activates the JNK pathway to initiate apoptosis. Inactivation of the *CDK5* and the *MEKK1* genes specifically suppressed apoptosis, without affecting other survival pathways of the UPR [48]. Although further investigation is needed to clarify the mechanisms by which ER stress activates CDK5, the CDK5-MEKK1-JNK pathway may also play an important role in ER stress-induced apoptosis in mammalian cells.

### 2.3. Signaling through PERK

PERK is a type I ER transmembrane protein with serine/threonine kinase activity in its *C*-terminal cytosolic domain, and PERK also recognizes the accumulation of misfolded proteins at its *N*-terminal luminal domain. The catalytic domain of PERK has the sequence similarity to the domains of other eIF2α family kinase members, such as PKR, general control non-derepressible-2 (GCN2), and heme-regulated inhibitor (HRI).

#### 2.3.1. Survival Signaling via PERK

Activation of PERK is triggered by the dissociation of BiP from the luminal domain of PERK, followed by PERK oligomerization and autophosphorylation [49]. Activated PERK phosphorylates eIF2α at Ser51, leading to the attenuation of global protein translation in order to reduce the protein load entering the ER [2]. In addition, phosphorylation of eIF2α induces the specific translation of ATF4 by changing the reading frame within its mRNA [50], after which ATF4 activates the transcription of many genes involved in functional UPR, including those associated with amino acid metabolism, redox homeostasis, and ER stress-induced apoptosis [51,52,53] (Figure 1). Therefore, the PERK-eIF2α-ATF4 pathway is also involved in dual biological functions: survival and apoptosis. However, *PERK^−/−^* cells and cells with a homozygous mutation of *eIF2α* at Ser51 (eIF2α S51A/S51A) have been shown to be sensitive to ER stress-induced apoptosis [50,52,54]. These observations suggest that PERK-mediated phosphorylation of eIF2α is necessary for the prevention of ER stress-induced apoptosis.

#### 2.3.2. Apoptosis Signaling via PERK

Under severe or prolonged ER stress conditions, activated PERK enhances the apoptosis-signaling pathway in a manner similar to that of IRE1α. In response to strong ER stress, the PERK-eIF2α-ATF4 pathway activates the transcription of proapoptotic factors, such as CHOP. CHOP is a member of the C/EBP family of bZIP transcription factors and upregulates a number of proapoptotic factors, including GADD34, death receptor 5 (DR5) [55], *tribbles*-related protein 3 (Trb3) [56], binding to microtubule (Bim) [57], and p53 upregulated modulator of apoptosis (PUMA) [58]. In particular, the CHOP-mediated induction of GADD34, which is a regulatory subunit of protein phosphatase 1 (PP1), leads to the dephosphorylation of eIF2α and finally to recovery from translational attenuation (Figure 2). Both *CHOP^−/−^* cells and *GADD34^−/−^* cells are defective in ER stress-induced apoptosis [59,60,61]. These data suggest that the PERK-eIF2α-ATF4 pathway converts survival signaling to apoptosis signaling through the expression of CHOP and GADD34.

## 3. ER-Associated Degradation

In the ER, folding, modification, and trafficking of secretory and transmembrane proteins are strictly regulated to maintain ER homeostasis. However, some proteins cannot be correctly folded as a result of a dysfunction of the folding system or an alteration within the gene itself. Cells utilize the ERAD pathway for eliminating these unfolded or misfolded (malfolded) proteins. In the ERAD pathway, malfolded proteins are recognized by chaperones, targeted to the retrotranslocon, dislocated from the ER to the cytosol, and degraded via the ubiquitin-proteasome system [62]. The ERAD pathway is conserved from yeast to mammals, and classified into the ERAD-C (cytosol), ERAD-L (lumen), and ERAD-M (membrane) pathways according to the particular site of structural defects in the degraded substrate proteins [63]. In yeast, the ERAD-C pathway is mediated by degradation of the Mat-α2-10 protein (Doa10p) complex, the ERAD-L pathway is mediated by the Hrd1p complex, and the ERAD-M pathway is mediated by an overlap of the activities of both Doa10p and Hrd1p. Doa10p and Hrd1p are ER membrane-associated E3 ligases, and each contains a cytosolic RING finger domain.

Ubiquitination of misfolded proteins via these E3 ligases is required for extraction of misfolded proteins by the cell division cycle protein 48 (Cdc48p) complex, which includes the Cdc48p ATPase and its cofactors, Npl4p and Ufd1p. In addition to Doa10p itself, the Doa10p complex is composed of Ubc6p and Ubc7p, which function as E2s in the ER membrane and the cytosol, respectively, and Cue1p and Ubx2p, which recruit the Ubc7p and the Cdc48p complex, respectively [63]. However, the Hrd1p complex is composed of Hrd1p, its binding partner Hrd3p, Der1p, Usa1p (which is necessary for Der1p recruitment), Ubc7-Cue1, Ubx2, the ER-resident HSP70 chaperone Kar2p, and luminal lectin Yeast osteosarcoma 9 (Yos9p), which is recruited by Hrd3p [63,64,65,66]. Furthermore, it has recently been reported that Htm1p/Mnl1p exhibits mannosidase activity and is involved in the recognition of unfolded glycoproteins upstream of Yos9p [67,68].

**Figure 3:**
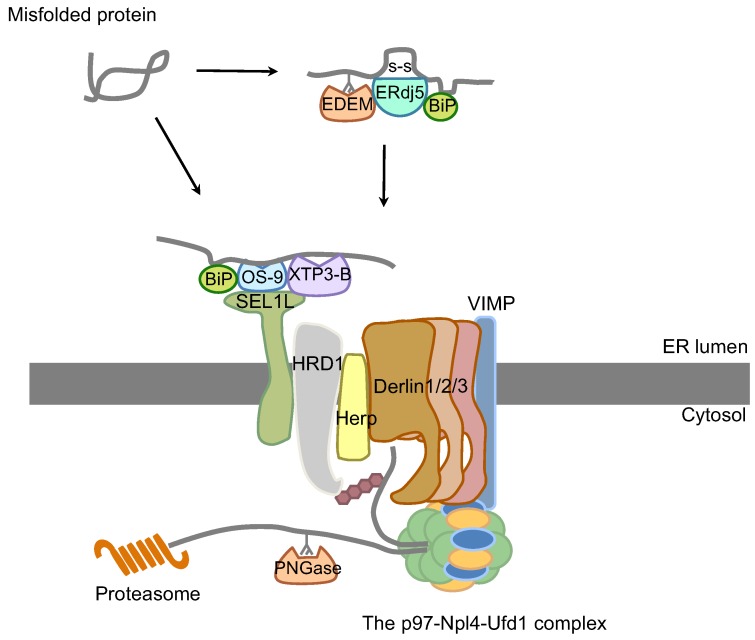
Mammalian ERAD: the HRD1 complex. ER luminal misfolded proteins are recognized by machinery including ER chaperone BiP, DnaJ family ERdj5, and lectins, such as ER degradation enhancing alpha-mannosidase-like protein (EDEM) family members, OS-9, and XTP3-B. Following its recognition, the terminally misfolded protein is recruited to the HRD1 complex via binding with SEL1L and is then brought to a putative retrotranslocon channel, which may include Derlin family proteins, HRD1, or the Sec61 complex. Finally, the protein is dislocated from the ER to the cytosol. Cytoplasm-exposed substrates are ubiquitinated by E3 ubiquitin ligase HRD1, and extracted by the p97-Npl4-Ufd1 complex anchored on the ER transmembrane through VIMP in an ATP-dependent manner. Finally, the extracted substrate is deglycosylated by PNGase, deubiquitinated, and degraded by the proteasome.

Although it has been reported that a variety of E3 ligases, including HRD1, gp78, RMA1/RNF5, TRC8, and TEB4 (Doa10p in yeast), can function in the mammalian ERAD pathway [69], the molecular mechanisms of the ERAD-C, ERAD-L, and ERAD-M pathways remain unclear. HRD1 is also known as synoviolin and functions as the E3 ligase for ubiquitination of substrates such as TCR-α, CD3-δ, and Parkin-associated endothelin-like receptors (Pael-R) [70,71,72]. In regard to the function of the HRD1 complex in the ERAD pathway (Figure 3), some misfolded glycoproteins are first recognized by ER degradation enhancing alpha-mannosidase-like protein (EDEM) family proteins (Htm1p/Mnl1p in yeast), which include EDEM1, EDEM2, and EDEM3 [73]. EDEM family proteins are α-mannosidase-like lectins that are induced by the UPR and bind to the misfolded glycoproteins with a mannose 8 structure [74,75,76]. Although EDEM3, but not EDEM1 or EDEM2, exhibits mannosidase activity, all of the EDEM family proteins play crucial roles in ERAD [73,77]. A recent study has shown that the ER-resident protein ERdj5 exhibits reductase activity, cleaves the disulfide bonds of misfolded proteins, and accelerates ERAD activity through its association with EDEM1 and BiP [78]. Moreover, in addition to misfolded glycoproteins, non-glycoproteins are also recognized by luminal lectins, such as osteosarcoma amplified 9 (OS-9) and XTP3-transactivated gene B protein (XTP3-B), which are mammalian homologs of Yos9p.

Misfolded proteins are subsequently transferred to the ER membrane-associated protein SEL1L, which functions as an adaptor for HRD1, and is stabilized through its interaction with HRD1. Following their recognition and targeting to the ERAD complex in the described system, substrates are dislocated into the cytosol through an unknown retrotranslocon. Although the Sec61 complex and Derlin family proteins (mammalian homologs of yeast Der1p) have been reported to be potential retrotranslocon candidates [79,80,81,82], the components of the retrotranslocon complex remain controversial. Although it is still unclear whether ER misfolded proteins can retrotranslocate through Sec61, the Sec61 translocon also functions as an ER calcium leak channel, which is sealed during resting conditions by BiP [83]. Under ER stress conditions, BiP is released, calcium subsequently leaks from the ER to the cytosol, and cells undergo apoptosis [84,85] (Figure 2). These observations suggest that in addition to the translocation of newly synthesized proteins, Sec61 may also contribute to maintaining ER calcium homeostasis. 

Recently, yeast Hrd1p has also been reported as a possible retrotranslocon because of requirements of its oligomerization for the ERAD-L pathway and its transmembrane domain for interaction with substrates [86]. Derlin family members each contain four transmembrane domains, and include Derlin-1, Derlin-2, and Derlin-3. They form homo- or hetero-oligomers and interact with many ERAD components, such as HRD1, SEL1L, Herp (mammalian homolog of yeast Usa1p), and VIMP (also known as an ER membrane receptor for p97/valosin-containing protein (VCP)) [86,87,88]. A recent study has illustrated that Derlin family proteins are inactive members of the rhomboid family of intramembrane proteases and play essential roles in retrotranslocation through conserved functional domains other than the catalytic domain [89]. Following retrotranslocation, substrates are ubiquitinated by the actions of HRD1 in the cytosol, extracted in an ATPase-dependent manner by the p97-Npl4-Ufd1 complex (the Cdc48p-Npl4p-Ufd1p complex in yeast), and deglycosylated by peptide *N*-glycanase (PNGase), which leads to degradation by the proteasome.

## 4. ER Stress and Related Diseases

Dysfunction of the UPR, or prolonged ER stress, disturbs ER homeostasis, leading to many human diseases, including neurodegenerative disease, metabolic disease, inflammatory disease, and diabetes mellitus. It is important to elucidate the mechanisms by which UPR signaling contributes to pathogenesis of these diseases. Elucidation of the molecular mechanisms of ER stress-related diseases may shed valuable light on these potential therapeutic targets. Table 1 summarizes the ER stress-related molecules associated with the diseases mentioned below.

### 4.1. Neurodegenerative Disease

In a large number of neurodegenerative diseases, the accumulation of misfolded proteins is a common and toxic feature; such proteins include amyloid β (Aβ), which produced by the processing of amyloid precursor protein (APP) in Alzheimer’s disease (AD); polyglutamine (polyQ), a product of CAG repeat expansions in polyQ diseases such as Huntington’s disease (HD); and the mutant form of superoxide dismutase 1 (SOD1), which is found in familial amyotrophic lateral sclerosis (ALS). In human pathogenic studies and animal models of several neurodegenerative diseases, the involvement of ER stress and the UPR signaling (e.g., the detection of ER stress markers, dysfunctional ERAD, and ER stress-induced apoptosis) has been reported.

**Table 1:** ER stress-related molecules associated with diseases.

Diseases	Key factors	The UPR signaling related physiological function & pathogenesis	References
*Neurodegenerative disease*			
Alzheimer’s disease	Amyloid β (Aβ), IRE1, XBP1, PERK, eIF2α, CHOP	- Aβ-induced activation of IRE1-XBP1 and PERK-eIF2α CHOP pathway	[90,91,92,93,94,95]
PolyQ diseases	Polyglutamine (polyQ), p97, IRE1, TRAF2, ASK1, JNK	- ERAD dysfunction by interaction of polyQ with p97	[96,97]
		- Proteasomal inhibition and activation of IRE1-TRAF2-ASK1-JNK pathway by polyQ	[7]
Amyotrophic lateral sclerosis	SOD1, Derlin-1, ASK1	- ERAD dysfunction and ASK1 activation by interaction of mutant SOD1 with Derlin-1	[98]
	XBP1	- Digestion of mutant SOD1 by XBP1-mediated autophagy	[99]
*Metabolic disease*			
Hypertriglyceridemia	CREBH	- CREBH-induced expression of lipid metabolism genes	[23,100]
*Inflammatory disease*			
Inflammatory bowel disease	IRE1β, XBP1	- Protective effects against intestinal inflammation by IRE1β and XBP1	[43,101]
*Diabetes mellitus*			
Type 1 diabetes	CHOP, NO	- NO-induced β cell apoptosis through ER stress-induced CHOP activation	[102]
Type 2 diabetes	PERK, eIF2α, ATF6	- Maintenance of ER function in β cells through PERK-eIF2α and ATF6	[50,54,103]
	CHOP	- Proapoptotic effects of CHOP in β cells	[61]
	XBP1	- Role of XBP1 in proinsulin processing and insulin secretion	[42]
	IRE1α	- IRE1α-induced *proinsulin* mRNA degradation under the condition of chronic high glucose exposure	[44,104]
Wolcott-Rallison syndrome	PERK	- Mutations of *PERK* related to β cell dysfunction in patients	[105]
Wolfram syndrome	WFS1 ATF6	- Mutations of *WFS1* in patients, Control of ER Ca^2+^ homeostasis by WFS1	[106]
		- Negative regulation of ATF6 and control in production and secretion of insulin by WFS1	[107,108]
*Cancer*			
Cancer	BiP, PERK, eIF2α, IRE1, XBP1	- Protective effects of BiP, the PERK-eIF2α pathway and the IRE1-XBP1 pathway in proliferation and progression of tumors	[109,110,111,112,113,114,115,116,117,118]
*Cardiovascular disease*			
Atherosclerosis	CHOP	- CHOP-induced Mφ apoptosis and plaque necrosis in atherosclerosis model mice	[119,120,121]

The familial AD-linked mutant forms of presenilin (PS) 1 that mediate cleavage of APP have been shown to induce elevated Aβ production. Mutant PS1 has been found to associate with IRE1 and inactivate IRE1, leading to the inhibition of UPR signaling [122]. In contrast, other groups have reported that the elevation of spliced *XBP1* mRNA, phosphorylated IRE1, and upregulated BiP expression were observed in the brain specimens of AD patients [90,91,92]. In cultured cells, the overexpression of XBP1s prevents Aβ-induced toxicity, whereas knockdown of *XBP1* accelerates Aβ-induced toxicity [123]. In addition, a study using cultured neuronal cells demonstrated that exogenous Aβ activates the PERK-eIF2α pathway, and the silencing of *PERK* by siRNA reduces Aβ-induced phosphorylation of eIF2α and enhances Aβ-induced cell death [93]. Moreover, in cultured cells and rodent brains, Aβ treatment induces CHOP expression [94,95], and reduction of CHOP by antisense RNA has been found to protect against Aβ-induced cell death [124]. These observations suggest that the UPR signaling pathways, including those of IRE1-XBP1 and PERK-eIF2α, may serve as therapeutic targets for AD. As for the involvement of ERAD, HRD1 enhances the ubiquitination and degradation of APP in cultured cells, resulting in a reduction of Aβ production [125]. Thus, the activation of the ERAD pathway may contribute to a decrease in the accumulation of the neurotoxic Aβ. 

In polyQ diseases, such as HD, polyQ associates with ERAD components, including p97, Npl4, and Ufd1, and disturbs their functions [96,97]. Furthermore, it has been shown that HRD1 accelerates the ubiquitination of polyQ and reduces the toxicity of polyQ [126]. In addition, polyQ inhibits proteasomal activity and thereby induces ER stress, leading to the activation of IRE1 and subsequently the TRAF2-ASK1-JNK pathway [7]. These results suggest that the ubiquitin proteasome system and the TRAF2-ASK1-JNK pathway may be potential therapeutic targets for polyQ disease. 

In ALS, deletion of *XBP1* results in delayed disease onset and extended life span due to enhanced autophagy [99]. Conversely, deletion of *PERK* causes enhanced disease progression and motoneuronal degeneration [127,128]. Moreover, various familial ALS-linked mutant SOD1 proteins have been found to associate with Derlin-1, but not Derlin-2 and -3, leading to dysfunction in ERAD, which induces ER stress and motoneuron death [98,129].

### 4.2. Metabolic Disease

The ER is a crucial site for not only for protein quality control, but also for lipid and glucose metabolism. Perturbations in ER homeostasis can result in the dysregulation of lipid and glucose metabolism in the liver and adipose tissue, leading to a number of metabolic diseases such as hepatic steatosis and dyslipidemia. ER stress is also known to contribute to lipogenesis and inactivate lipoprotein secretion [130]. Overexpression of BiP inhibits the insulin-induced activation of sterol regulatory element binding protein 1c (SREBP1c), a key transcription factor in lipogenesis, and results in the palliation of hepatic steatosis in mice [131]. ATF6 interacts with SREBP2 and CREB-regulated transcription coactivator 2 (CRTC2), both of which are ER transmembrane-localized regulators of lipid metabolic genes. These interactions inhibit the subsequent expression of lipogenic and gluconeogenic genes [132,133]. CREBH (an ER transmembrane-localized bZIP transcription factor similar to ATF6) is specifically expressed in restricted tissues, including the liver (see Section 2.1). CREBH activation by ER stress induces the expression of lipid metabolic genes [23,133]. *CREBH^−/−^* mice fed with atherogenic high-fat diets develop profound hepatic steatosis and hypertriglyceridemia [100]. The PERK-eIF2α pathway also controls lipogenesis through the function of SREBP1 as a transcription factor for lipid metabolic genes [134].

Although the IRE1α-XBP1 pathway induces the expression of the lipogenic transcription factor C/EBPα and promotes lipogenesis [135], CHOP inversely disturbs the function of C/EBPα and inhibits lipogenesis [136]. Similarly, XBP1s directly induces the expression of many lipogenic genes. Moreover, the regulatory subunits of phosphatidyl inositol 3-kinase (PI3K), p85α and p85β, form heterodimers, which dissociate upon insulin treatment. This dissociation allows p85 to interact with XBP1s and increase its extent of nuclear translocation, suggesting that the UPR signaling may be upregulated during metabolic overload [137,138]. Interestingly, a liver-specific deletion of *XBP1* inhibits steatosis as well as insulin resistance in the liver [139] and results in marked hypocholesterolemia and hypotriglyceridemia [140]. Furthermore, it has been reported that the silencing of lipid metabolic genes through IRE1α-mediated mRNA decay leads to a decrease in plasma lipids in mice [141]. It has recently been shown that the IRE1-XBP1 pathway induces PDI expression to increase microsomal triglyceride-transfer protein (MTP) activity for hepatic very low-density lipoprotein (VLDL) assembly and lipid homeostasis [142]. These findings suggest that the ER stress signaling pathways maintain lipid and glucose homeostasis through either the upregulation or the downregulation of the UPR.

### 4.3. Inflammatory Disease

Numerous recent studies have revealed that the UPR pathways contribute inflammatory signaling to assist in the recovery from tissue damage. In inflammatory diseases, such as inflammatory bowel disease, the ER stress-induced inflammation exacerbates disease progression. Under ER stress, IRE1α activates the JNK-AP1 pathway and the IκB kinase (IKK)-NFκB pathway as well as the splicing of XBP1. The PERK-eIF2α pathway activates the IKK-NFκB pathway through the translational attenuation of IκB. In addition, the PERK-eIF2α pathway induces the transcriptional activation of ATF4, while ATF6 activates the Akt-NFκB pathway. These transcription factors (XBP1, AP1, NFκB, and ATF4) induce the expression of pro-inflammatory cytokines, such as tumor necrosis factor (TNF)-α, interleukin-1 (IL-1), IL-6, IL-8, and monocyte chemoattractant protein 1 (MCP1) [130,143].

In inflammatory bowel disease, IRE1-XBP1 has been shown to function protectively. Deletion of the *IRE1β* or *XBP1* gene in mouse intestinal epithelium leads to an increased susceptibility to dextran sulfate sodium (DSS)-induced colitis [43,101]. Deletion of *XBP1* in the mouse intestine also results in intestinal inflammation [101]. These observations suggest that the IRE1-XBP1 pathway may contribute to the maintenance of the intestinal epithelium.

There have been several reports describing the significance of the UPR pathway in innate immunity in *Caenorhabditis* (*C.*) *elegans*. The role of the p38 MAPK pathway is conserved in innate immunity from *C. elegans* to humans [144]. During *C. elegans* larval development, PMK-1 (a p38 ortholog) is activated by *Pseudomonas aeruginosa* and activates the IRE1-XBP1 pathway [145]. The infection of a loss-of-function *XBP1* mutant with *P. aeruginosa* leads to a disruption in ER morphology and larval lethality [145]. These observations strongly suggest that XBP1 plays an essential role in protecting the host through the activation of innate immunity responses.

### 4.4. Diabetes Mellitus

In pancreatic β cells, protein synthesis is dramatically increased in response to glucose stimulation. Thus, β cells must be strictly regulated by the UPR so that translated proinsulin will be correctly folded in the ER. However, the activation of prolonged or excessive ER stress results in β cell dysfunction and may lead to type 2 diabetes. To date, numerous reports have shown that the PERK-eIF2α pathway in β cells is a key pathway for maintaining the ER environment during proinsulin synthesis [50,54,103]. In patients with Wolcott-Rallison syndrome, which is characterized by the neonatal or early onset of insulin-requiring diabetes, mutations in the *PERK* gene have been identified [105]. Furthermore, *PERK^−/−^* mice are prone to hyperglycemia and diabetes, due to a reduction in insulin secretion [50,146]. Because some groups have reported that polymorphisms and haplotypes of *ATF6α* are related to type 2 diabetes, ATF6α also appears to be important for the function of β cells [147,148,149]. A recent study has suggested that ATF6α protects β cells from ER stress [150].

In addition to the aforementioned genetic evidence from human diseases and knockout mice, UPR is also activated in β cells derived from patients with type 1 and type 2 diabetes [151,152,153]. Moreover, it has been shown that the nutrient stimulation of rat islets moderately activates the UPR in a manner dependent on protein synthesis, while exerting complex effects on ER stress signaling in β cells [154]. These findings suggest that the UPR plays several roles in β cell function. Nitric oxide (NO) produced by cytokines also induces the depletion of ER Ca^2+^ store and ER stress, leading to β cell death. This phenomenon has been related to type 1 diabetes. CHOP has been reported to play a central role in the NO-induced apoptosis of β cells [102]. Deletion of *ASK1* or *CHOP* delays the onset of diabetes in heterozygous Akita mice that carry a conformation-altering missense mutation (Cys96Tyr) in insulin 2 [12,155]. Moreover, in several type 2 diabetes mouse models, deletion of *CHOP* has been shown to not only prevent β cell apoptosis but also improve β cell function through the induction of UPR target genes, antioxidative stress genes, as well as the inhibition of proapoptotic genes [61]. These observations suggest that CHOP may be a key player linking the accumulation of misfolded proteins to oxidative stress and apoptosis in β cells, under the conditions of increased insulin demand [61].

β cell-specific mice defective in *XBP1* exhibit hyperglycemia and glucose intolerance due to decreased insulin secretion from their β cells [42], suggesting that XBP1 may be required for glucose-stimulated proinsulin processing and insulin secretion. Chronic exposure of β cells to high glucose causes ER stress and hyperactivation of IRE1α, leading to the degradation of IRE1α-mediated *proinsulin* mRNA [44,104,156]. Thus, IRE1α signaling is important for regulating insulin biosynthesis, and further studies are needed to clarify the physiological functions of mRNA degradation by IRE1α.

Over 100 mutations in the *Wolfram syndrome 1* (*WFS1*) gene have been identified in patients with Wolfram syndrome, which is characterized by juvenile diabetes and optical atrophy [157]. WFS1 is an ER transmembrane protein induced by XBP1 under ER stress, and regulates ER calcium homeostasis, thereby promoting β cell survival [106]. A recent study has shown that WFS1 negatively regulates ATF6α under normal conditions and deletion of *WFS1* induces chronic UPR signaling through prolonged ATF6α activation, leading to β cell apoptosis [107]. Moreover, it has been reported that WFS1 translocates to the plasma membrane following glucose stimulation, interacts with adenylyl cyclase 8 (AC8), controls production of cAMP, and ultimately regulates the production and secretion of insulin [108].

Under normal conditions, insulin receptors activated by insulin subsequently induce cytosolic tyrosine phosphorylation of insulin receptor substrate-1 (IRS-1). In obesity, ER stress promotes the IRE1-JNK-dependent serine phosphorylation of IRS-1, which in turn inhibits the insulin receptor-induced tyrosine phosphorylation of IRS-1 [158]. These UPR signaling pathways make major contributions to type 1 and type 2 diabetes mellitus and maintain ER homeostasis and β cell function.

### 4.5. Cancer

Tumor cells are challenged by microenvironments such as hypoxia and hypoglycemia, which lead to the induction of ER stress [159]. It is well known that the UPR pathways are activated during tumor cell growth [1]. BiP is induced in numerous tumor cells and contributes to proliferation and survival of tumor cells [109]. Suppression of BiP expression inhibits tumor cell growth, progression, and metastasis in both *in vitro* and *in vivo* models [110,111,112]. Overexpression of BiP partially suppresses apoptosis of human gastric cancer cells induced by celecoxib, a non-steroidal anti-inflammatory drug (NSAID), and the knockdown of *BiP* dramatically enhances apoptosis [113]. In addition, the PERK pathway has also been reported to play a critical role in tumor cell survival [114]. Inactivation of the PERK pathway by either mutations in the kinase domain of PERK or a mutation of eIF2α at its phosphorylation site impairs survival under hypoxic conditions. PERK also promotes cancer cell proliferation and tumor growth by limiting oxidative DNA damage [115]. In a rodent glioma model, the inhibition of the IRE1 pathway suppressed tumor cell growth and angiogenesis [116]. Deletion of *XBP1* was found to increase sensitivity to hypoxia-induced cell death and reduce tumor formation [117,118]. These observations suggest that the IRE1-XBP1 pathway may also play an important role in tumor growth. The UPR pathway may serve as a potential target for the treatment of cancer.

### 4.6. Cardiovascular Disease

Oxidative stress, hypoxia, and increased protein synthesis have been demonstrated in cases of heart failure and lead to the induction of ER stress. In patients with heart failure, the elevation of spliced *XBP1* mRNA and increased BiP expression are observed, suggesting that UPR activation is associated with the pathophysiology of heart failure [160,161,162]. In these patients, the increased expression of *ATF4* and *CHOP* mRNAs is also observed [163]. Furthermore, ubiquitinated proteins are accumulated in failing human hearts [164,165]. Because the inhibition of the ubiquitin proteasome system triggers ER stress, an accumulation of unfolded proteins may also cause UPR in diseased hearts. Although further investigation is needed to clarify the mechanisms by which ER stress contributes to the pathogenesis of heart failure, the UPR pathway may be a potential target for disease treatment. Atherosclerosis is the most common pathological process underlying cardiovascular disease. In atherosclerosis, chronic ER stress activates inflammatory signaling in macrophages. Deletion of *CHOP* reduces apoptosis in macrophages and plaque necrosis, leading to suppression of atherosclerotic progression in both *ApoE^−/−^* and *Ldlr^−/−^* mice, which are two common animal models of atherosclerosis [119,120,121]. These findings strongly suggest that CHOP is an accelerating factor in the development of arteriosclerosis. Further research is needed to elucidate the contribution of each UPR pathway to cardiovascular disease.

### 4.7. Therapeutic Approach

A large number of studies have focused on therapeutic approaches utilizing chemical chaperones to stabilize misfolded proteins, improve the ER folding capacity and suppress ER retention of these misfolded proteins. 4-Phenyl butyric acid (4-PBA) and taurine-conjugated ursodeoxycholic acid (TUDCA) are effective chemical chaperones in several diseases, including inflammatory bowel disease, atherosclerosis, and type 2 diabetes. 4-PBA and TUDCA have been shown to reduce inflammatory stimuli-induced ER stress in cultured intestinal epithelial cells, as well as DSS-induced acute and chronic colitis in mice [166]. Moreover, 4-PBA reduces lipotoxicity-induced ER stress in macrophages and apoptosis in atherosclerotic lesions [167]. In a mouse model of type 2 diabetes, 4-PBA and TUDCA reduced ER stress and restored glucose homeostasis [168]. In contrast, 4-PBA has been reported to inhibit histone deacetylases (HDACs) at high concentrations [169]. In order to ensure that these chemical compounds are applicable to disease treatments, further studies will be needed to elucidate their side effects and evaluate their safety. 

Several studies have focused on survival signaling in the UPR, such as signaling of the PERK-eIF2α pathway. Salubrinal is a compound that inhibits dephosphorylation of eIF2α, delays the recovery of protein translation, and protects cells from ER stress [170,171]. Salubrinal abates IFN-γ-induced oligodendrocyte loss and hypomyelination [172]. In familial ALS, salubrinal attenuates the disease manifestation and delays its progression [104]. Salubrinal also has therapeutic potential in other neurodegenerative diseases: it has been reported to reduce Aβ-induced neurotoxicity in cultured neuronal cells [95], inhibit the protein aggregation and cell death caused by polyQ [173], reduce the ER accumulation of α-synuclein, and extend life span in a mouse model of α-synucleinopathies, including PD [174]. However, opposing effects of salubrinal on β cells in type 2 diabetes have been reported [175]. Salubrinal induces a marked eIF2α phosphorylation and potentiates the inhibitory effects of free fatty acids on protein synthesis and insulin release. This selective activation of the PERK-eIF2α pathway, but not of the IRE1 and ATF6 pathways, leads to a marked induction of ATF4 and CHOP, resulting in β cell apoptosis. The molecular mechanisms and *in vivo* effects of salubrinal remain controversial, and further studies are needed to support the applicability of salubrinal as a therapeutic agent.

It is difficult to develop therapeutic approaches specifically targeting ER stress because UPR signaling exerts dual biological functions related to both survival and apoptosis. Moreover, the particular organs or cells involved in specific diseases must receive their therapeutic effects selectively and accurately. Despite these difficulties, one possible therapeutic approach may involve activating the IRE1α pathway in a manner that does not induce JNK activation but induces the splicing of *XBP1* mRNA and upregulation of functional UPR target gene transcription, including those of chaperones involved in recovering the ER capacity, without inducing apoptotsis signaling.

## 5. Concluding Remarks

Under ER stress, cells recognize perturbations of ER homeostasis and direct the UPR signal to either survival or apoptosis pathways, depending on the intensity of ER stress. Survival pathways such as ATF6, IRE1α-XBP1, PERK-eIF2α-ATF4, and ERAD restore the ER capacity through transcriptional activation of genes related to the refolding and maturation of misfolded proteins. However, prolonged or severe ER stress directs cell death through an apoptosis pathway such as IRE1-ASK1-JNK or PERK-eIF2α-ATF4-CHOP. Under these physiological states, it is important to clarify the cross-talk among these UPR pathways from the perspectives of duration and amplitude. In contrast, dysfunction of the UPR pathway, or chronic ER stress, may contribute to the pathogenesis of numerous human diseases. Therefore, further investigations of the molecular mechanisms involved in the regulation of UPR signaling may lead to the discovery of novel therapeutic targets.
